# Relationship between the expression of cyclooxygenase-2 and survivin in primary pterygium

**Published:** 2009-02-27

**Authors:** Cristina Maxia, Maria Teresa Perra, Paolo Demurtas, Luigi Minerba, Daniela Murtas, Franca Piras, Renè Cabrera, Domenico Ribatti, Paola Sirigu

**Affiliations:** 1 Department of Cytomorphology, University of Cagliari Medical School, Cagliari, Italy; 2 Department of Public Health, University of Cagliari Medical School, Cagliari, Italy; 3 Department of Ophthalmology, University of Cuenca Medical School, Cuenca, Ecuador; 4 Department of Human Anatomy and Histology, University of Bari Medical School, Bari, Italy; 5 Instituto del Cancer ‘Solca’, Cuenca, Ecuador

## Abstract

**Purpose:**

To investigate the expression of cyclooxygenase-2 (COX-2) in a group of 93 Ecuadorian primary pterygia and to evaluate a possible association between COX-2 and survivin.

**Methods:**

Primary pterygium samples were treated for the immunohistochemical evaluation of COX-2 and survivin. Mouse monoclonal antibody to COX-2 and rabbit polyclonal antibody to survivin were used. Statistical analysis was performed using the SPSS statistical software package, version 15.0.

**Results:**

In our study, 63 (67.7%) primary pterygia samples were positive for COX-2 staining, and 70 (75.3%) specimens were positive for survivin expression. In the group of pterygia with survivin immunostaining, there were 55 (78.6%) samples with COX-2 expression. The staining of both COX-2 and survivin was localized in the lower and middle layers of the epithelium. When analyzed by Fisher's exact test, the expression of COX-2 showed a strong significant correlation with survivin (p=0.0002).

**Conclusions:**

These data, showing a significant correlation between COX-2 and survivin in primary pterygium, suggest that pterygium may originate through an anti-apoptotic mechanism.

## Introduction

Pterygium is a common conjunctival disorder exhibiting degenerative and hyperplastic changes as well as proliferative, inflammatory features and a rich vasculature. Although extracellular matrix remodeling [1], inflammatory process [2,3], anti-apoptotic mechanisms [4,5], cytokines [6], growth and angiogenic factors [7,8], and viral infection [9,10] have been proposed as causative agents in its pathogenesis, several investigators consider pterygium an ultraviolet radiation (UV)-related disease [11-16]. Pterygium has long been considered as a chronic degenerative condition; however, because of the finding of abnormal expression of p53 protein in the epithelium [17-21], pterygium has been considered an UV-related tumor rather than a degenerative disease. To support this hypothesis, we reported in a previous study the presence of conjunctival melanocytic pigmented lesions in pterygium and, among these, two primary acquired melanosis with atypia [22].

UV irradiation has a key role in the formation of reactive oxygen species (ROS), short-lived entities continuously generated at low levels during the course of normal aerobic metabolism, which have been associated with inflammation, initiation, and progression of tumors through activation of carcinogens [23]. ROS can induce cyclooxygenase 2 (COX-2) production [24]. COXs are rate-limiting enzymes involved in prostanoid synthesis, which convert arachidonic acid into prostaglandin (PG) H2, a substrate for specific prostaglandin synthases [25]. Two isoforms of COX, encoded by separate genes, have been isolated, cloned, and sequenced. The isoform designated as COX-1, constitutively expressed in most cells and tissues, modulates normal physiologic responses such as regulation of renal and vascular homeostasis and gastroprotection. The other one, designed as COX-2, is rapidly induced by growth factors, cytokines, hormones, hypoxia, bacterial endotoxins, tumor promoters, and UV light, and its protein is frequently undetectable at baseline in most normal adult tissues. Both ROS and COX-2 play an important role in UV-related cutaneous carcinogenesis [26]. Subbaramaiah et al. [27] reported that COX-2 expression is suppressed by normal p53, suggesting that loss of p53 function may result in the induction of COX-2 expression. Consistent overexpression of COX-2 was observed in a broad range of premalignant, malignant, and metastatic human epithelial cancers [28].

Aziz and co-authors [29] demonstrated that UV-B exposure leads to a significant activation of survivin, a member of the inhibitor of apoptosis protein family (IAPs) that is overexpressed in most human malignancies and implicated in the cellular stress response. It is well known that the functional loss of wild-type p53 is associated with the upregulation of survivin expression in several types of tumors. Recently, we have demonstrated in pterygium a significant association between survivin expression and oxidative stress and p53, and we have hypothesized a UV-related loss of normal p53 functionality [5].

Since several studies demonstrated a correlation between COX-2 and survivin expression in tumors [30-32], the aim of the present study was to investigate the expression of COX-2 in a group of 93 Ecuadorian primary pterygia by immunohistochemistry and to evaluate a possible association between COX-2 and survivin.

## Methods

### Patients and study design

This study was based on an analysis of formalin-fixed, paraffin embedded primary pterygia harvested from 93 patients (36 males and 57 females) in a cluster of 111 people that underwent excision by bare sclera technique at the Department of Pathology at the Cancer Center of SOLCA (Cuenca, Ecuador) from July 2004 to December 2007. The selected patients were of mixed race, between American Indian and Hispanic. Ages ranged between 18 and 77 years (mean age 43.35±13.9). Thirty-five patients lived in the countryside and 58 in an urban setting. All the selected patients were outdoor workers. Most of the lesions were located on the nasal side and only the head of primary pterygium was used as the pterygium sample. Pterygium morphology was clinically graded as atrophic (24 cases), intermediate (53 cases), or fleshy (17 cases) according to an assessment of pterygium translucency. Normal conjunctiva samples as controls were collected from medial bulbar conjunctiva of 10 patients (six males and four females) without pterygium and pinguecula who underwent cataract surgery. Ages ranged between 25 and 70 years (mean age 52.1±16.02). The younger patients were surgically treated for traumatic cataract. Seven patients lived in the countryside and three resided in an urban setting. Relevant clinical features of the patients are summarized in Table 1. Patients did not receive any medication before surgery except for a topical anesthetic, and no drugs or chemical agents were used during surgical operation. The study protocol was approved by the local research ethic committee, and informed consent was obtained from all subjects according to the World Medical Association Declaration of Helsinki. Complete information on patients was available in all cases.

**Table 1:** Clinical features of the patients.

**Samples**	**Variables**	**Number of patients**
**Pterygium**		
	**Gender**	
	Male	36
	Female	57
	**Age**	
	≤ 43*	47
	> 43*	46
	**Residency**	
	Urban	58
	Rural	35
	**COX-2 expression**	
	Positive	63
	Negative	30
	**Survivin expression**	
	Positive	70
	Negative	23
**Conjunctiva**		
	**Gender**	
	Male	6
	Female	4
	**Age**	
	< 55*	5
	≥55*	5
	**Residency**	
	Urban	3
	Rural	7

This table presents clinical variables of the study’s participants and the immunohistochemical analysis. The asterisk indicates the median value.

### Immunohistochemistry

Microtome histological sections (6–7 μm thick) were treated for the immunohistochemical demonstration of COX-2 and survivin using the streptavidin-biotin alkaline phosphatase method. They were dewaxed in xylene and rehydrated in a graded alcohol series and phosphate-buffered saline (PBS). Water-bath, heating-based antigen retrieval was performed by immersion in 10 mM citrate buffer solution (pH 6.0) at 95 °C for 40 min. After gradual cooling for 20 min, sections were treated for 45 min with 10% normal goat or normal horse serum in PBS. Mouse monoclonal antibody (1:100, clone 4H12; Novocastra, Newcastle upon Tyne, UK) to human COX-2 raised against amino acids 30–163 of the NH_2_-terminus of human COX-2 molecule and rabbit polyclonal antibody (1:1000; Novus Biologicals, Littleton, CO) to recombinant human survivin protein, which recognizes the full-length recombinant human survivin, were used as primary antisera and incubated for 60 min at room temperature while biotinylated anti-mouse and anti-rabbit IgG were used as secondary antisera (1:200; Vector Laboratories, Burlingame, CA) and incubated for 30 min at room temperature. The sections were further incubated in alkaline phosphatase streptavidin (1:1000; Vector Laboratories) for 30 min at room temperature and reacted with Fast Red Substrate System (Dako, Glostrup, Denmark). All sections were thoroughly rinsed in PBS between each step, and finally counterstained with Mayer hematoxylin and mounted in glycerol gelatin (Sigma, St. Louis, MO).

Sections of Crohn’s disease were used as positive control tissue for COX-2, while sections of human cutaneous malignant melanoma were used as positive control for survivin. Negative controls were obtained by omission of the primary antibody or by replacing the primary antibody with an isotype-matched antibody. Positive and negative controls were run simultaneously.

Micrographs were captured by a digital camera Canon PowerShot A620, (Canon Inc., Tokyo, Japan) on a microscope Zeiss Axiophot (Carl Zeiss Inc., Oberkochen, Germany), and processed by Adobe Photoshop (version 7.0; Adobe Systems, Inc., San Jose, CA) software.

### Evaluation of immunoreactivity

Results were independently evaluated by three observers in a blinded fashion. Four to six 200X fields covering almost the whole of each of the four sections per sample were examined with a 144-intersection point square reticulum (0.78 mm^2^) inserted in the eyepiece and scored for the percentage of epithelial immunoreactive cells. The cutoff level for immunohistochemical analysis was set at 10%, meaning that those samples with more than 10% of cells stained were considered to be positive. Patients were divided into four groups, COX-2-positive, COX-2-negative, survivin-positive, and survivin-negative.

### Statistics

The results were assessed with Fisher’s exact test. Data were computed by the SPSS statistical software package, version 15.0 (SPSS Inc., Chicago, IL). A p value of less than 0.05 was considered statistically significant.

## Results

### COX-2 expression

**Figure 1:**
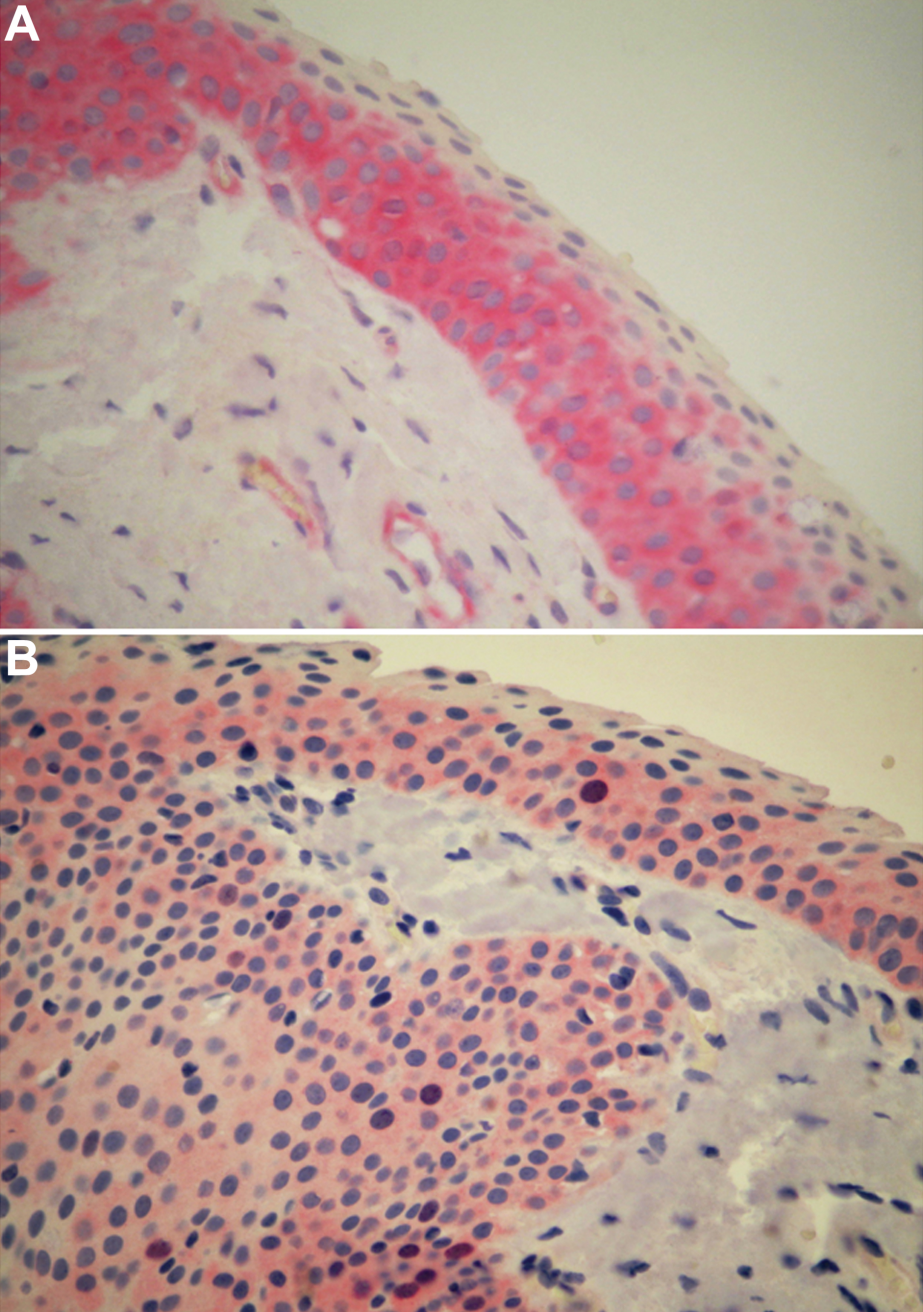
Immunohistochemical staining for COX-2 and survivin in adjacent sections of primary pterygium. The expression of both COX-2 (**A**) and survivin (**B**) was localized in the lower and middle layers of the epithelium. The immunoreactivity for survivin was localized to the nuclei and cytoplasm, while COX-2 expression was limited to cytoplasm. Note in **A**, COX-2-immunoreactive endothelial cells in the lamina propria. Each section was counterstained with hematoxylin. Original magnification; 400X.

**Figure 2:**
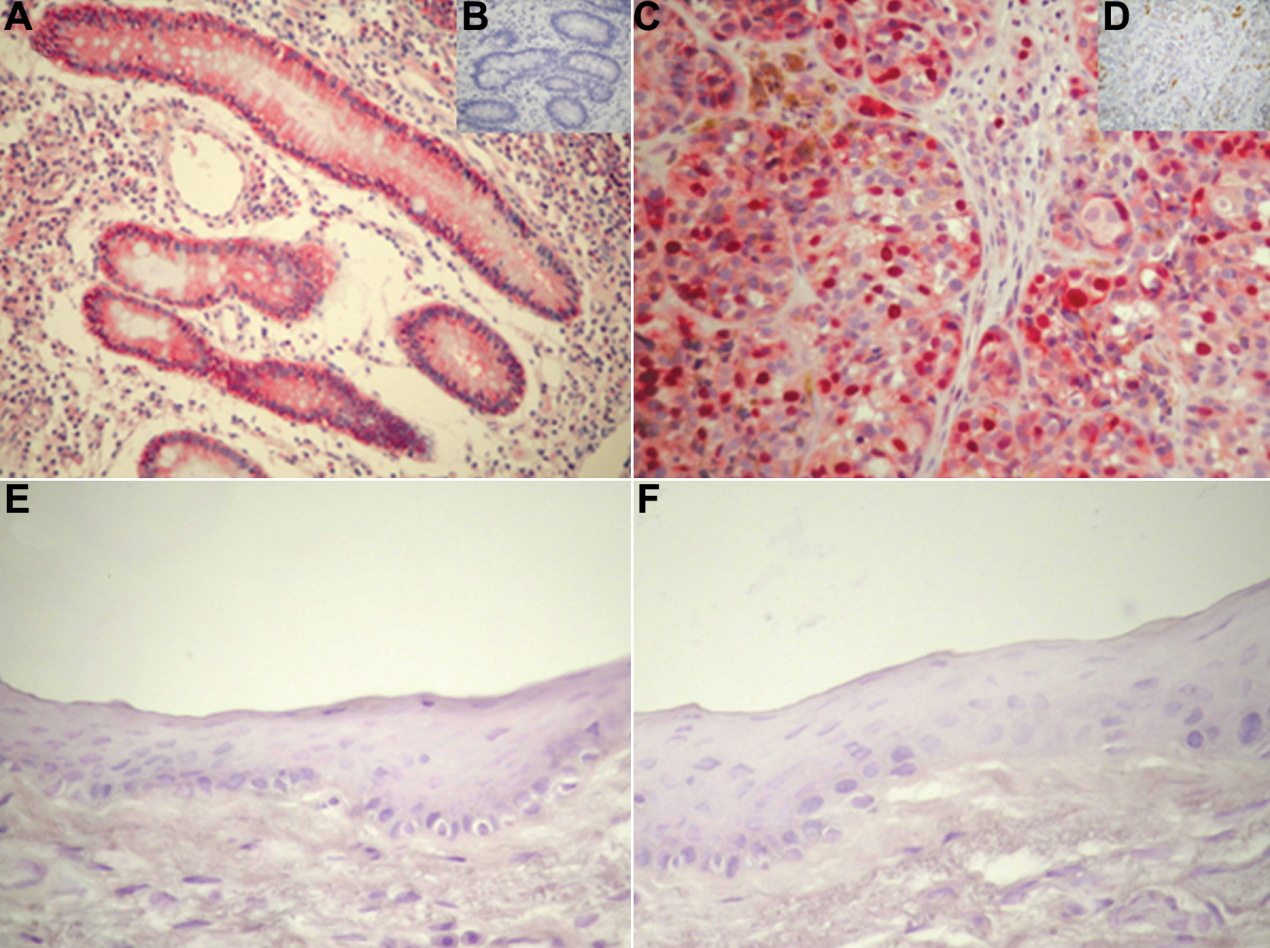
Immunohistochemical staining for COX-2, and survivin in control sections. Sections from Crohn’s disease (**A**) and human cutaneous malignant melanoma (**C**) were included as positive controls. No immunostaining for COX-2 (**E**) and survivin (**F**) in normal conjunctiva was observed. Sections incubated without a primary antibody (inset **B**) or with an isotype control antibody (inset **D**) displayed no immunoreactivity. Each section was counterstained with hematoxylin. Original magnification, **A**: 200X; **C**,**E**,**F**: 400X; insets **B**,**D**: 400X.

In our study, 63 (63/93, 67.7%) primary pterygia samples were positive for COX-2 staining. The staining was detectable in the cytoplasm of cells localized to the basal and middle layers of the epithelium and in the endothelial cells of microvessels (Figure 1A). No immunostaining for COX-2 in normal conjunctiva was present (Figure 2E). Positive control section from Crohn’s disease demonstrated immunoreactivity for COX-2 in the cytoplasm of both epithelial cells located in the crypts, surface epithelial cells, and mononuclear cells of the lamina propria (Figure 2A). No staining was detected when the primary antibody was omitted (Figure 2B) or when an isotype control antibody was applied.

### Survivin expression

Positive staining was detected in 70 (70/93, 75.3%) primary pterygia. The immunoreactivity was localized to the nuclei and cytoplasm of the epithelial cells (Figure 1B). No substantial staining was visible in the subepithelial fibrovascular layers. No immunostaining for survivin in normal conjunctiva was detectable (Figure 2F). The positive control section from human cutaneous melanoma demonstrated immunoreactivity for survivin in the nuclei and cytoplasm of tumoral cells (Figure 2C). Reactivity was absent in sections incubated without primary antibody, and no reactivity developed when the isotype control antibody was used (Figure 2D).

### Relationship between COX-2 and survivin

The relationship between COX-2 and survivin is shown in Table 2. In the group of pterygia positive to survivin, 55/70 (78.6%) samples showed COX-2 expression. When analyzed by Fisher's exact test, the expression of total survivin showed a strong significant correlation with COX-2 (p=0.0002).

**Table 2:** Relationship between COX-2 and survivin**.**

**Group of patients**	**Survivin positive**	**Survivin negative**	**Total**
**COX-2 positive**	55	8	63
**COX-2 negative**	15	15	30
**Total**	70	23	93

Shown is the relationship between COX-2 and survivin. In the group of pterygia with survivin immunostaining, there were 55 (55/70, 78.6%) samples with COX-2 expression. Survivin expression was significantly associated with COX-2 positivity (p=0.0002 assessed by Fisher’s exact test).

## Discussion

UV light is one of the most important factors involved in the pathogenesis of pterygium, but the mechanism by which UV radiation induces this disease still remains unknown. Several studies demonstrated that absorption of UV by molecules in the cell results in the generation of ROS, which can cause oxidative DNA damage, often in the form of 8-oxo- or 8-hydroxy-deoxyguanosine (8-OHdG) adducts [33,34]. In keratinocytes, this DNA damage leads to an increase in p53 expression [35], which arrests the cell cycle at G_1_/S and drive the cell through apoptosis if the damage cannot be repaired. This mechanism prevents the accumulation of potentially oncogenic DNA mutations. Therefore, chronic or multiple UV-B exposure can lead to p53 mutation and/or allelic loss [36,37] or a UV-induced defect in p53 activation [20]. Previously, we have observed p53 overexpression in 8-OHdG immunoreactive pterygia, providing evidence that pterygium is a tumor-like growth disorder related to faulty apoptosis [21].

Extensive data has validated the role of UV-B as both a tumor initiator and promoter probably by its ability to upregulate COX-2 expression, which converts arachidonic acid into PGH2 [25]. These products may act as tumor promoters in UV-initiated tissue or they may enhance initiation due to their ability to act as oxidants [38]. Subbaramaiah et al. [27] reported that normal p53 suppresses COX-2 expression, suggesting that loss of p53 function may result in the induction of COX-2 expression. COX-2 stimulates tumor cell proliferation and increases the invasiveness of malignant cells [39] and enhances angiogenesis through the production of vascular endothelial growth factor (VEGF). Finally, a stable overexpression of COX-2 determines a dramatic resistance to UV-induced apoptosis [40].

Herein, we have used immunohistochemistry to demonstrate COX-2 expression in 67.7% of pterygial specimens in the cytoplasm of keratinocytes localized in the lower and middle layers of the epithelium whereas no immunoreactivity was detectable in normal conjunctiva. Our data are in agreement with Chiang et al. [12], who have demonstrated COX-2 expression in primary pterygium. The results support the causal relationship between COX-2 and pterygium and provide molecular evidence of the effects of UV radiation in this lesion.

Since 1979 [41], it has been proposed that pterygium could represent a precancerous condition of the mucosal epithelium, analogous to cutaneous actinic keratosis for histologic features [42]. Actinic keratoses are dysplastic lesions considered to be precursor to squamous cell carcinomas, which result from chronic exposure to sunlight. Buckman and co-authors [38] demonstrated the prevalence of COX-2 not only in UV-induced human squamous cell carcinoma but also in actinic keratoses.

Aziz et al. [29] have demonstrated that UV-B exposure leads to a significant activation of survivin, a protein belonging to the inhibitor of apoptosis (IAP) gene family, in human epidermal keratinocytes. Survivin is expressed in embryonic or proliferating normal adult tissues and highly upregulated in almost all types of human malignancy [43]. Survivin overexpression inhibits extrinsic or intrinsic apoptotic pathways through an inhibition of caspases [44]. Previously, we have demonstrated for the first time an upregulation of survivin expression and a relationship between survivin, oxidative stress, and p53 overexpression in primary pterygia [5]. We then hypothesized that the cooperation between survivin and UV-induced functional loss of p53 could be responsible for aberrant inhibition of apoptosis in pterygium. In the present study, we have demonstrated a strong significant COX-2 expression in survivin immunoreactive epithelial cells. This is the first study to report a significant correlation between COX-2 upregulation and survivin expression in primary pterygium. Several authors already reported a relationship between COX-2 and survivin expression in other kinds of malignant lesions such as ovarian [32], breast [31], and non-small cell lung cancer [30]. Our results are in agreement with previous data by Erkanly et al. [45] about the positive correlation of the two proteins not only in the endometrial carcinoma but also in a premalignant lesion such as endometrial hyperplasia. Overall, these data indicate that COX-2 may be involved in pterygium growth by increasing the activation of the anti-apoptotic protein, survivin. Moreover, the relationship between COX-2 and survivin suggest a potential origin of pterygium through an anti-apoptotic mechanism and confirm that this lesion may develop toward a neoplastic-like growth disorder instead of a degenerative condition of the conjunctiva. These evidences provide useful information toward the development of novel therapeutic strategies for pterygium involving the use of COX-2 and survivin inhibitors alone or in combination, which might contribute to the treatment of pterygium through the suppression of the anti-apoptotic effect of survivin and the tumorigenic induction of COX-2.
